# A national cohort study of melanoma *BRAF* status, testing patterns, patient and tumour characteristics, treatment and survival in England from 2016 to 2021

**DOI:** 10.1093/bjd/ljaf351

**Published:** 2025-09-03

**Authors:** Khaylen Mistry, Polly Jeffrey, Nick J Levell, Oliver Kennedy, Kathryn Richardson, Paul Craig, Chloe Bright, Siobhan Taylor, John Ragan, Dimitrios Karponis, Joanna Pethick, Katrina Lavelle, Fiona McRonald, Steven Hardy, Sally Vernon, Paul Lorigan, Zoe C Venables

**Affiliations:** Department of Dermatology, Norfolk and Norwich University Hospitals Foundation Trust, Norwich, UK; Norwich Medical School, University of East Anglia, Norwich, UK; National Disease Registration Service, NHS England, Leeds, UK; National Disease Registration Service, NHS England, Leeds, UK; Department of Dermatology, Norfolk and Norwich University Hospitals Foundation Trust, Norwich, UK; Norwich Medical School, University of East Anglia, Norwich, UK; Department of Oncology, The Christie NHS Foundation Trust, Manchester, UK; University of Manchester, Manchester, UK; Norwich Epidemiology Centre, University of East Anglia, Norwich, UK; Departments of Cellular Pathology, North Bristol NHS Trust, Bristol, UK and Gloucestershire Hospitals NHS Foundation Trust, Cheltenham, UK; National Disease Registration Service, NHS England, Leeds, UK; Department of Cellular Pathology, Gloucestershire Hospitals NHS Foundation Trust, Cheltenham, UK; Patient and Public Representative, UK; Department of Dermatology, Norfolk and Norwich University Hospitals Foundation Trust, Norwich, UK; Norwich Medical School, University of East Anglia, Norwich, UK; National Disease Registration Service, NHS England, Leeds, UK; National Disease Registration Service, NHS England, Leeds, UK; National Disease Registration Service, NHS England, Leeds, UK; National Disease Registration Service, NHS England, Leeds, UK; National Disease Registration Service, NHS England, Leeds, UK; Department of Oncology, The Christie NHS Foundation Trust, Manchester, UK; University of Manchester, Manchester, UK; Department of Dermatology, Norfolk and Norwich University Hospitals Foundation Trust, Norwich, UK; Norwich Medical School, University of East Anglia, Norwich, UK; National Disease Registration Service, NHS England, Leeds, UK

## Abstract

**Background:**

Inadequacy of testing for melanoma *BRAF* status results in delayed access to systemic therapy. *BRAF* mutations and their association with patient/tumour characteristics and survival is poorly understood.

**Objectives:**

To report national data from England on the frequency of molecular BRAF testing; the association of patient/tumour characteristics with *BRAF* mutations; and the treatment and survival of patients with *BRAF* mutations.

**Methods:**

This national retrospective cohort study identified all new melanomas and molecular BRAF testing in England diagnosed from 2016 to 2021 using population-based data from the National Disease Registration Service. Multivariate logistic regression determined the association between *BRAF* testing with patient/tumour characteristics and *BRAF* genotype with patient/tumour characteristics. Age-standardized net survival analysed melanoma-specific mortality by *BRAF* genotype.

**Results:**

Of new cases of melanoma, 14% (*n* = 13 138/91 415) had a *BRAF* test registered. The proportion of successfully tested tumours that were *BRAF*-mutated was 34% (*n* = 4424/13 012). The West Midlands tested the highest proportion of cutaneous tumours (23%; *n* = 1783/7901) vs. the lowest in Yorkshire and the Humber (11%; *n* = 856/7760). Female patients [odds ratio (OR) 0.82, 95% confidence interval (CI) 0.79–0.86] and those aged > 80 years (OR 0.88, 95% CI 0.83–0.93) were less likely to be tested for *BRAF* mutations. *BRAF* mutations were associated with female gender (OR 1.16, 95% CI 1.07–1.26). Patients aged > 80 (OR 0.36, 95% CI 0.32–0.40) had lower odds of having *BRAF*-mutated tumours. Patients with *BRAF* mutations had a lower 5-year net survival [55.9% (95% CI 52.7–59.2) vs. *BRAF* wildtype 5-year net survival 62.2% (95% CI 60.1-64.5)], particularly in stage II disease.

**Conclusions:**

This study presents the largest dataset on national melanoma *BRAF* status published to date. The data highlight geographical and demographic variations in *BRAF* testing and the impact of *BRAF* mutations on survival rates, particularly in patients with stage II disease. This highlights the critical role of consistent, early and accurate testing to ensure equal care, guide treatment decisions and understand prognosis.

## Teaser

This national cohort study presents the largest dataset on melanoma *BRAF* biomarker status published to date, with 13 138 *BRAF*-tested tumours registered. Variations in *BRAF* testing rates by geographical and demographic factors are identified, advocating for improved evidence-led policies on *BRAF* testing to ensure consistent and equitable patient care. Five-year net survival is lower in patients with *BRAF* mutations, particularly in those with stage II melanoma, emphasizing the importance of integrating *BRAF* testing into early-stage melanoma management.

Linked Article: Whiteman *Br J Dermatol* 2025; **193**:1041.

What is already known about this topic?

- In clinical practice, *BRAF* mutations are the most important known oncogenic ‘drivers’ of melanoma.
- *BRAF* mutations are prognostically significant and predictive of response to targeted therapy and immunotherapy.
- Inadequacy of testing for *BRAF* status in melanoma results in delayed access to systemic therapy.
- *BRAF* mutations and their association with patient and tumour characteristics and survival in melanoma is poorly understood; few national datasets have been published globally.

What does this study add?

- This study presents the largest dataset on melanoma BRAF biomarker status ever published, with 13 138 *BRAF*-tested tumours registered.
- Variations in *BRAF* testing rates by geographical and demographic factors were identified, advocating for improved evidence-led policies on *BRAF* testing to ensure consistent and equitable patient care.
- Five-year net survival was lower in patients with *BRAF* mutations, particularly in those with stage II melanoma, emphasizing the importance of integrating *BRAF* testing into early-stage melanoma management.

The most important ‘driver’ mutation of melanoma is a mutation in *BRAF*. *BRAF* mutations are prognostically significant and predictive of response to targeted therapy and immunotherapy in the adjuvant and palliative settings.^1^ In 2022, the National Institute for Health and Care Excellence (NICE) published national recommendations to perform *BRAF* analysis for stage IIB–IV melanoma and to consider analysis for stage IIA.^2^ Prior to 2022, although national guidance existed on who should receive systemic therapy, with adjuvant targeted therapy approved in 2018, there was no guidance on which patients should be *BRAF* tested in England.^2^ Since 2013, international guidance and expert consensus have recommended routinely determining *BRAF* status in stage III/IV melanoma, and some recommendations include stage II.^2–6^  *BRAF* testing methodology has changed, as per expert consensus and national survey data from England. Prior to 2022, *BRAF* status was determined by polymerase chain reaction (PCR) testing and, to a lesser extent, immunohistochemistry (IHC), and not next-generation sequencing (NGS).^6, 7^ Inadequacy of testing for *BRAF* mutations may result in underdetection of mutations and delayed access to or underutilization of systemic therapy.

The frequency of *BRAF* mutations and their association with pathological and demographic factors is an understudied topic, with international variation.^8–13^ Previous epidemiology studies have been limited by small cohorts, lack of data on covariates and pooling data from various cancers.^8^ Improved understanding of the characteristics associated with *BRAF* mutations may identify risk factors to support a targeted approach to testing. The identification of patients at high risk of melanoma-related death based on their *BRAF* genotype can inform recommendations for treatment, follow-up and eligibility for adjuvant trials.

No national data from England on the molecular genetics of melanoma have been reported and few national datasets have been published globally. The aim of this study was to report on diagnoses of melanoma in England between 2016 and 2021 on: (i) the frequency of molecular BRAF testing, including regional variations; (ii) the association of patient and tumour characteristics with *BRAF* mutations; and (iii) the treatment received and survival of patients with *BRAF* mutations.

## Materials and methods

### Study design, data sources and variables

This national retrospective cohort study used English cancer registry data from the National Disease Registration Service (NDRS). The registry maintains details of all cancers diagnosed each year across England (population 56 489 800 according to the 2021 census).^14^ It is mandatory for all National Health Service (NHS) pathology laboratories, and recommended for all private pathology laboratories in England, to provide all cancer pathology reports to the dataset. Only a small proportion of melanomas are managed privately in England and NDRS data are considered the gold-standard dataset for cancer data representation. Pathology report data are combined with Patient Administration System and Cancer Outcomes and Services Dataset to form the National Cancer Registration Dataset (NCRD). The NDRS receives and records data on molecular genetic testing (including *BRAF* mutational analysis) directly from genomics or molecular pathology laboratories across England. IHC testing for BRAF-V600E mutations is included in pathology report text from pathology laboratories, but these IHC tests are not routinely recorded as discrete data items, so are less amenable to analysis.

Patient records within the NCRD were linked to the National Radiotherapy Dataset, Systemic Anti-Cancer Therapy dataset, NHS Hospital Episode Statistics datasets and death registrations from the Office for National Statistics. These linked data sources are considered gold standard, providing information on genetics and treatment (systemic therapy, radiotherapy, surgery), with the years of data included considered complete and high quality.^15–17^ Data were extracted on 1 February 2024 for new melanomas diagnosed from 1 January 2016 to 31 December 2021, which was the latest available year.

Melanoma was identified using International Classification of Diseases (ICD)-10 site codes and ICD-O-3 morphology and behaviour codes (Table S1; see Supporting Information).^18^ Disease-specific death was defined by the ICD-10 code C43 or C80 (melanoma skin cancer, malignant neoplasm without specification of site). Cause of death and vital status of patients was determined until 31 December 2021.

Patient variables extracted included age at diagnosis, gender, ethnicity, tumour site, stage at diagnosis, geography and deprivation quintile. Ethnicity was self-reported. Table S2 and Figure S1 describe how variables were defined (see Supporting Information). RECORD guidelines were adhered to (https://www.record-statement.org).

### Outcomes and statistical analysis

Data were extracted using SQL Developer^©^ 19.4.0.354.1759 (Oracle, Austin, TX, USA). Statistical analyses were done using R 4.3.2^©^ (R Foundation for Statistical Computing, Vienna, Austria) and STATA 18.0^©^ (StataCorp, Cary, NC, USA).

Multivariate logistic regression adjusted for age, gender, site, self-reported ethnicity, deprivation quintile and geographical region was used to determine the association between *BRAF*-tested cutaneous tumours tested within 180 days of diagnosis and stage at diagnosis. In this analysis those not tested within 180 days were considered not tested on diagnosis. A *BRAF* test within 180 days of diagnosis was considered an acceptable timeframe for the test to be requested upon initial diagnosis rather than recurrence. For subsequent analyses the definition of *BRAF* testing was not restricted by the requirement for a *BRAF* test within a specified time of the melanoma diagnosis date.

Multivariate logistic regression examined the association between *BRAF* testing of cutaneous melanoma and covariates, to understand any potential selection bias. To examine gaps in the recorded molecular data, a random sample of 650 pathology reports, including 50 pathology reports from each region that mentioned ‘BRAF’, were reviewed to ascertain the proportion that underwent BRAF IHC testing.

All data after *BRAF* testing patterns were conditioned on the subset of tumours with a confirmed *BRAF* test. Multivariate logistic regression was used to examine the association between *BRAF* genotype and the covariates. Each covariate was built into the regression model in steps with testing for interactions between covariates. The variance inflation factor was checked to ensure minimal multicollinearity. Stratification by gender was examined, as anatomical site of melanoma and age at diagnosis differ by gender.

Survival time was calculated for patients diagnosed with their first melanoma in England between 2016 and 2020. Patients were censored at death, loss to follow-up (loss to NHS through lack of updated information or emigration) or the study end date (31 December 2021). Age-standardized net survival at 5 years was calculated by comparing overall survival in the melanoma cohort with age (single year), year, gender, deprivation quintile and geography lifetables.^19,20^ Hazard ratios (HRs) for disease-specific mortality were estimated using multivariate Cox regression models with a second model including systemic anticancer therapy, with time to melanoma-specific death as the outcome. The proportional hazards assumption was examined for each covariate using log–log plots. Sensitivity analysis to evaluate immortal time bias compared the HRs from the multivariate Cox models starting from the diagnosis date and *BRAF* testing date (cohort restricted to pathological diagnosis date – BRAF test date ≤ 90 days).

## Results

### *BRAF* testing patterns

Of new melanomas diagnosed, 14% (*n* = 13 138/91 415) had a *BRAF* test registered and 1% (*n* = 126/13 138) had failed, inconclusive or unknown test results. The proportion of successfully tested tumours that were *BRAF* mutated was 34% (*n* = 4424/13 012). Of the successfully tested tumours, 96% (*n* = 12 490/13 012) were cutaneous. *BRAF* testing of cutaneous melanomas increased from 2016 to 2019 then decreased from 2020 to 2021; there was no regional variation in this trend across England (Table S3; see Supporting Information).

The percentage of stage III/IV cutaneous tumours that were *BRAF* tested was 52% (*n* = 4558/8731), whereas the percentage of stage II and I tumours tested was 26% (*n* = 4441/16 844) and 3% (*n* = 1366/52 353), respectively. The percentage of *BRAF*-tested cutaneous tumours that were tested within 180 days of diagnosis was 78% (*n* = 9720/12 490). Compared with stage II melanoma, those with stage III [odds ratio (OR) 0.35, 95% confidence interval (CI) 0.31–0.39] or IV disease (OR 0.11, 95% CI 0.08–0.15) were less likely to have a *BRAF* test after 180 days, whereas those with stage I disease (OR 2.00, 95% CI 1.75–2.27) were more likely to have had a test (Table S4; see Supporting Information).

The West Midlands region tested the highest proportion of cutaneous tumours (23%; *n* = 1783/7901) vs. the lowest in Yorkshire and the Humber (11%; *n* = 856/7760) (Table 1). Regional stage breakdown revealed that 10% (*n* = 812/7901) of cutaneous tumours in the West Midlands were stage III/IV vs. 12% (*n* = 927/7760) in Yorkshire and the Humber (Table S5; see Supporting Information). The same proportion of melanomas (6%; *n* = 3/50) underwent BRAF IHC testing from the West Midlands and Yorkshire and the Humber (Table 1). Female patients (OR 0.82, 95% CI 0.79–0.86) and patients aged > 80 years (OR 0.88, 95% CI 0.83–0.93) were less likely to have had *BRAF* testing (Table S6; see Supporting Information). The percentage of pathology reports that underwent BRAF IHC testing was 6% (*n* = 36/650). For melanomas with known mutation subtype following molecular BRAF testing, 74% (*n* = 2099/2821) were V600E (Table S7; see Supporting Information).

**Table 1:** BRAF testing patterns for cutaneous melanoma diagnosed between 2016 and 2021 by geographical region in England

Region	Cutaneous tumours registered in region (*n*)	Proportion of cutaneous tumours successfully *BRAF* tested	Proportion of cutaneous tumours that failed *BRAF* testing	Proportion of cutaneous tumours not *BRAF* tested	Proportion of stage III/IV cutaneous tumours successfully *BRAF* tested	Proportion of successfully tested cutaneous tumours *BRAF* mutated	Proportion of tumours IHC tested^a^
London	7205	854/7205 (12)	20/874 (2)	6331/7205 (88)	244/618 (39)	306/854 (36)	4/50 (8)
East of England	9950	1375/9950 (14)	19/1394 (1)	8556/9950 (86)	625/1214 (51)	505/1375 (37)	5/50 (10)
North-East	5265	625/5265 (11.9)	3/628 (0.5)	4637/5265 (88.1)	239/437 (54.7)	251/625 (40.2)	1/50 (2)
North-West	12 296	2390/12 296 (19)	5/2395 (< 1)	9901/12 296 (81)	1038/1359 (76)	834/2390 (35)	3/50 (6)
Yorkshire and the Humber	7760	856/7760 (11)	13/869 (1)	6891/7760 (89)	433/927 (47)	298/856 (35)	3/50 (6)
East Midlands	7065	956/7065 (14)	5/961 (< 1)	6104/7065 (86)	321/630 (51)	355/956 (37)	2/50 (4)
West Midlands	7901	1783/7901 (23)	9/1892 (< 1)	6109/7901 (77)	564/812 (69)	582/1783 (33)	3/50 (6)
South-East	17 648	1986/17 648 (11)	33/2019 (2)	15 629/17 648 (89)	465/1499 (31)	680/1986 (34)	0/50 (0)
South-West	12 363	1665/12 363 (13)	15/1680 (< 1)	10 683/12 363 (86)	629/1235 (51)	590/1665 (35)	1/50 (2)
Total	87 453	12 490/87 453 (14)	122/12 612 (1.0)	74 841/87 453 (86)	4558/8731 (52)	4401/12 490 (35)	22/450 (5)

Data are presented as *n* (%). IHC, immunohistochemistry. ^a^From a random sample of 450 pathology reports (50 from each region) that mentioned ‘BRAF’.

### Patient and tumour characteristics by *BRAF* genotype

*BRAF*-mutated genotypes were associated with female gender (OR 1.16, 95% CI 1.07–1.26) (Table 2). Patients aged > 80 years (OR 0.36, 95% CI 0.32–0.40) and those who self-reported in NDRS as Black, Asian, Mixed or Other (OR 0.65, 95% CI 0.47–0.89) had lower odds of having *BRAF* mutations. Advanced-stage melanoma had the highest odds of being *BRAF* mutated [stage III OR 1.61 (95% CI 1.46–1.78); stage IV OR 1.47 (95% CI 1.27–1.70)]. For cutaneous melanoma, head/neck tumours had the lowest odds of being *BRAF* mutated (OR 0.43, 95% CI 0.38–0.48). Stratification by gender resulted in no marked differences in results (Table S8; see Supporting Information).

**Table 2:** Odds ratios (ORs) for the association between covariates and mutated BRAF genotype

Variable	WT (*n* = 8089; 65%)	Mutated (*n* = 4401; 35%)	Total (*n* = 12 490)	Logistic regression, univariate analysis (*n* = 12 490), OR^a^ (95% CI)	Logistic regression, multivariate analysis (*n* = 12 490), OR^a^ (95% CI)
Gender					
Male	4850 (60)	2494 (57)	7344 (59)	Ref.	Ref.
Female	3239 (40.0)	1907 (43.3)	5146 (41.2)	**1.14 (1.06–1.23)**	**1.17 (1.07–1.26)**
Age group (years)					
< 70	3295 (41)	2785 (63)	6080 (49)	Ref.	Ref.
70–79	2582 (32)	1053 (24)	2775 (22)	**0.48 (0.44–0.53)**	**0.51 (0.47–0.56)**
≥ 80	2212 (27)	563 (13)	3635 (29)	**0.30 (0.27–0.33)**	**0.36 (0.32–0.40)**
All sites^b^					
Skin	8089 (94)	4401 (99)	12 490 (96)	Ref.	Ref.
Mucosal	386 (4)	14 (< 1)	400 (3)	**0.07 (0.04–0.11)**	**0.07 (0.04–0.11)**
Ocular	79 (1)	3 (< 1)	82 (< 1)	**0.07 (0.02–0.19)**	**0.06 (0.01–0.16)**
Other	34 (< 1)	6 (< 1)	40 (< 1)	**0.26 (0.10–0.57)**	**0.26 (0.10–0.58)**
Skin site					
Head/neck	1763 (22)	552 (13)	2315 (19)	**0.34 (0.30–0.38)**	**0.43 (0.38–0.48)**
Lower limb	1921 (24)	1063 (24)	2984 (24)	**0.60 (0.54–0.66)**	**0.59 (0.53–0.66)**
Upper limb	1595 (20)	613 (14)	2208 (18)	**0.41 (0.37–0.46)**	**0.43 (0.39–0.49)**
Trunk	1849 (23)	1715 (39)	3564 (29)	Ref.	Ref.
Overlapping/unknown/external genitals	961 (12)	458 (10)	1419 (11)	**0.51 (0.45–0.58)**	**0.49 (0.43–0.57)**
Ethnicity					
White	7615 (94)	4144 (94)	11 759 (94)	Ref.	Ref.
Black/Asian/Mixed/Other^c^	141 (2)	60 (1)	201 (2)	0.78 (0.57–1.05)	**0.65 (0.47–0.89)**
Black	31 (< 1)	5 (< 1)	36 (< 1)	–	–
Asian	44 (< 1)	10 (< 1)	54 (< 1)	–	–
Mixed	11 (< 1)	6 (< 1)	17 (< 1)	–	–
Other	55 (< 1)	39 (< 1)	94 (< 1)	–	–
Unknown	333 (4)	197 (4)	530 (4)	1.09 (0.91–1.30)	0.99 (0.82–1.20)
Deprivation quintile					
1 (most deprived)	1018 (13)	615 (14)	1633 (13)	**1.18 (1.04–1.34)**	1.10 (0.96–1.25)
2	1289 (16)	724 (16)	2013 (16)	1.10 (0.98–1.24)	1.04 (0.91–1.17)
3	1781 (22)	959 (22)	2740 (22)	1.05 (0.95–1.18)	1.04 (0.93–1.17)
4	1954 (24)	1058 (24)	3012 (24)	1.06 (0.95–1.18)	1.05 (0.94–1.18)
5 (least deprived)	2047 (25)	1045 (24)	3092 (25)	Ref.	Ref.
Disease stage					
I	812 (10)	554 (13)	1366 (10.9)	**1.75 (1.54–1.98)**	**1.42 (1.24–1.62)**
IA	187 (2)	116 (3)	303 (2)	–	–
IB	599 (7)	431 (10)	1030 (8)	–	–
IX (stage I but not specified if IA/B)	26 (< 1)	7 (< 1)	33 (< 1)	–	–
II	3194 (39.5)	1247 (28.3)	4441 (36)	Ref.	Ref.
IIA	666 (8)	317 (7)	983 (8)	–	–
IIB	1178 (15)	401 (9)	1579 (13)	–	–
IIC	1329 (16)	525 (12)	1854 (15)	–	–
IIX (stage II but not specified if IIA/B/C)	21 (< 1)	4 (< 1)	25 (< 1)	–	–
III	1879 (23)	1455 (33)	3334 (27)	**1.98 (1.80–2.18)**	**1.61 (1.46–1.78)**
IIIA	163 (2)	201 (5)	364 (3)	–	–
IIIB	413 (5)	280 (6)	693 (6)	–	–
IIIC	797 (10)	542 (12)	1339 (11)	–	–
IIID	52 (< 1)	45 (1)	97 (< 1)	–	–
IIIX (stage III but not specified if IIIA/B/C/D)	454 (6)	387 (9)	841 (7)	–	–
IV	763 (9)	461 (10)	1224 (10)	**1.55 (1.35–1.77)**	**1.47 (1.27–1.70)**
IVC	1 (< 1)	0	1 (< 1)	–	–
IVX	762 (9)	461 (10)	1223 (10)	–	–
Unknown	1441 (18)	684 (16)	2125 (17)	**1.22 (1.09–1.36)**	**1.23 (1.08–1.40)**
Region					
London	548 (7)	306 (7)	854 (7)	1.04 (0.88–1.23)	1.14 (0.95–1.35)
East of England	870 (11)	505 (11)	1375 (11)	1.08 (0.94–1.24)	1.13 (0.97–1.30)
North-East	374 (5)	251 (6)	625 (5)	**1.25 (1.04–1.50)**	**1.25 (1.04–1.52)**
North-West	1556 (19)	834 (19)	2390 (19)	Ref.	Ref.
Yorkshire and the Humber	558 (7)	298 (7)	856 (7)	1.00 (0.85–1.17)	0.99 (0.83–1.17)
East Midlands	601 (7)	355 (8)	956 (8)	1.10 (0.94–1.29)	1.16 (0.99–1.37)
West Midlands	1201 (15)	582 (13)	1783 (14)	0.90 (0.79–1.03)	0.92 (0.80–1.05)
South-East	1306 (16)	680 (15)	1986 (16)	0.97 (0.86–1.10)	**1.17 (1.02–1.34)**
South-West	1075 (13)	590 (13)	1665 (13)	1.02 (0.90–1.19)	1.13 (0.98–1.30)
Management					
Definitive surgery	7151 (88)	3914 (89)	11 065 (89)	–	–
Systemic therapy	2369 (29)	1859 (42)	4228 (34)	–	–
Radiotherapy	707 (9)	363 (8)	1080 (9)	–	–
Systemic therapy^d^					
Immunotherapy only	3285 (42)	732 (17)	4017 (33)	–	–
Targeted therapy only	38 (< 1)	1037 (24)	1075 (9)	–	–
Targeted and immunotherapy	44 (< 1)	728 (17)	772 (6)	–	–
Neither	4454 (57)	1801 (42)	6255 (52)	–	–

Data are presented as *n* (%). CI, confidence interval; WT, wildtype. Bold denotes a statistically significant result (i.e. *P* < 0.05) ^a^ORs are for *BRAF* mutated (reference BRAF WT). Multivariate model includes gender, age, self-reported ethnicity, cutaneous site only, deprivation quintile, stage and geographical region. ^b^A separate model for all sites (including mucosal, ocular and other). For this separate model, the total was 13 012 as it included noncutaneous tumours. ^c^See Table S2 for a full breakdown of self-reported ethnicities in the National Disease Registration Service. ^d^For systemic therapy, the total was 12 119 as counted at a patient level.

### Systemic anticancer treatment received by *BRAF* genotype

Of patients with stage III/IV *BRAF*-mutated melanoma, 20% (*n* = 372/1896) received immunotherapy only, 31% (*n* = 583/1896) received targeted therapy only, 20% (*n* = 383/1896) received immunotherapy and targeted therapy, and 29% (*n* = 558/1896) received neither immunotherapy nor targeted therapy (Table S9; see Supporting Information). Of patients with stage III/IV *BRAF*-mutated melanoma who received systemic anticancer therapy, 59% (*n* = 784/1338) received targeted therapy as the first-line treatment.

### Survival by *BRAF* genotype

**Figure 1:**
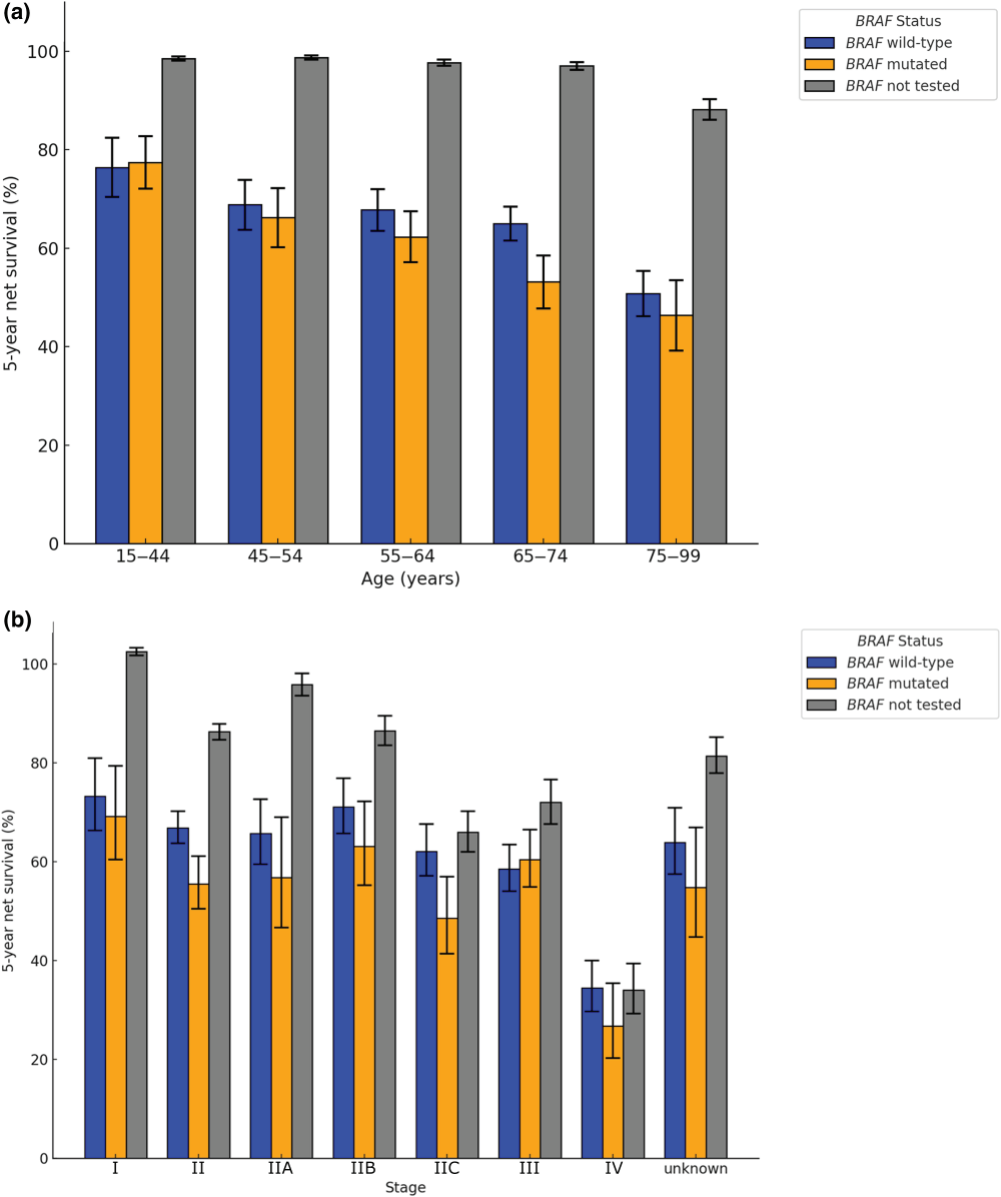
Age-standardized net survival at 5 years calculated by comparing overall survival from diagnosis date in patients with cutaneous melanoma between 2016 and 2020 with an age-, year-, gender-, deprivation quintile- and geography-matched general population cohort. Variance bars represent 95% confidence intervals. Counts per category are provided in Table S10. (a) Net survival by age and (b) net survival by melanoma stage.

*BRAF*-mutated tumours had a lower net survival [*BRAF* wildtype 5-year net survival 62.2% (95% CI 60.1-64.5) vs. *BRAF*-mutated 5-year net survival 55.9% (95% CI 52.7–59.2)], particularly in stage II disease [BRAF wildtype 5-year net survival 66.8% (95% CI 63.7–70.2) vs. BRAF-mutated 5-year net survival 55.5% (95% CI 50.5–61.1)] [Figure 1; Table S10 (see Supporting Information)]. *BRAF*-mutated genotype was associated with higher disease-specific mortality (HR 1.19, 95% CI 1.10–1.29) (Table S11; see Supporting Information). HRs measuring survival from the diagnosis date or the *BRAF* testing date were similar, confirming minimal immortal time bias (HR 1.18, 95% CI 1.06–1.31) (Table S12; see Supporting Information). Of patients with *BRAF* mutations treated with systemic anticancer therapy, those who received first-line immunotherapy had lower disease-specific mortality (HR 0.36, 95% CI 0.29–0.45) than those treated with first-line targeted therapy (Table S13; see Supporting Information).

## Discussion

This study presents the largest dataset on national melanoma BRAF biomarker status published to date, and the most complete reporting on testing patterns, and characteristics and survival associated with *BRAF* genotype.

We found that 35% (*n* = 4401/12 490) of cutaneous tumours tested positive for *BRAF* mutations, which was lower than reported in the existing literature (approximately 40%).^8^ Approximately half of patients with stage III/IV melanoma had a *BRAF* molecular test registered, suggesting inadequate testing over the study period. The observed increase in *BRAF* testing in 2019 may be explained by the approval of adjuvant targeted therapy in 2018. The decrease in testing from 2020 may be explained by the COVID-19 pandemic and shorter duration of follow-up. Our study identified variation in *BRAF* testing by geographical regions in England. Exploratory analyses revealed that regional differences in stage at diagnosis or IHC testing did not explain testing patterns; however, future research should further explore IHC testing by region. Regional variation and low *BRAF* testing rates in patients with stage III/IV melanoma may be explained by the lack of clear guidance on testing during the cohort period, regional differences in population characteristics or more selective testing, but they most likely reflect *BRAF* testing methods and variation in compliance in sending data between regional laboratories. NICE recommendations for *BRAF* testing were released in 2022 and may reduce future variation in clinical practice; however, adherence to guidance should be regularly audited and in this rapidly changing field of melanoma biomarker and therapeutic advancement, national guidance must remain up to date to improve melanoma prognosis and equity of care.^2^ Although these regional variations are not generalizable to other countries, we expect that these differences may be even greater where there is more restricted healthcare provision.^21^

*BRAF*-mutated genotype was associated with female gender, younger age, cutaneous site and the trunk, in keeping with the current literature.^8,22^ Despite the reported greater risk of *BRAF* mutations in female patients, these patients were less likely to be *BRAF* tested, which may be explained by the fact that female patients tend to present with earlier-stage melanoma.^22^ There is a paucity of data on the association of *BRAF* genotype with ethnicity, largely because previous studies have primarily been reported from populations comprised mainly of White patients.^8^ This study identified that *BRAF*-mutated genotypes were associated with White patients, but the findings should be interpreted with caution due to low numbers of people of other ethnic groups, the higher relative proportion of acral/subungual/mucosal melanoma in other ethnic groups with the absence of data on histological subtype, and the heterogenous nature of the cohort of the patients belonging to various ethnic groups. Stage III/IV melanoma had the highest odds of being *BRAF* mutated. Stage I melanoma had higher odds of being *BRAF* mutated than stage II melanoma. Given that guidance does not routinely recommend *BRAF*-testing stage I melanoma, this could be explained by *BRAF* testing on recurrence and targeted testing. Staging data reflected stage at diagnosis only, with no validated marker for recurrence. The time between the pathological diagnosis and *BRAF* testing was longer for early-stage melanoma, which suggested a high proportion of the stage I tumours were tested on recurrence.

*BRAF*-mutated genotype was associated with lower survival, particularly in stage II melanoma. Survival by *BRAF* status in stage I, III and IV tumours had overlapping CIs, but cohort numbers were smaller for these groups, and all but stage III tumours showed a trend towards worse outcomes in *BRAF*-mutated tumours. Similarly, a systematic review of 52 studies, representing 7519 patients, found that *BRAF* mutations were associated with reduced overall survival (HR 1.23, 95% CI 1.09–1.38).^23^ The review highlighted the paucity of evidence concerning the prognostic role of *BRAF* status in early-stage melanoma.^23^ With current international guidance mostly recommending *BRAF* testing in stage III/IV melanoma and consideration of testing in stage II, this study supports determining *BRAF* status in stage II melanoma as it could offer key information with the potential to contribute to prognostic scores and inform treatment, follow-up and clinical trials.^2,4^ The majority of patients with stage III/IV *BRAF*-mutated melanoma treated with systemic anticancer therapy received targeted therapy as their first line of treatment. Of patients with *BRAF*-mutated melanoma, those who received first-line immunotherapy had higher disease-specific survival; however, limitations of the data included difficulty identifying treatment intent, recurrence and treatment windows. Recently published SECOMBIT and DREAMseq trial data reported that first-line immunotherapy has a survival benefit, which may account for some of the differences seen in survival by *BRAF* status.^24,25^ In the period 2016–21 there was no adjuvant systemic anticancer therapy approved for stage I/II melanoma, so patients who received systemic anticancer therapy may have had *BRAF* testing of an archival primary tumour following relapse and should not be considered as having had stage I/II melanoma at the time of testing. Interpreting the downstream effects of *BRAF* mutations, especially by stage and systemic therapy use, is challenged by the accuracy of stage recording in national data and the changing landscape of systemic therapy, with the approval of adjuvant BRAF/MEK inhibitors for stage III melanoma in 2018, which may have confounded the dataset.

The main strength of this study is the size and quality of the data; however, the key issue is that national datasets may not capture data on *BRAF* testing and downstream analyses well. Therefore, national datasets may not be best placed to assess patterns of *BRAF* testing and draw inferences from these. Limitations included regional differences in the completeness of molecular data submissions, primarily affecting London and the Thames Valley region. There may have been changing practices to molecular testing over time; however, given the negligible differences in the sensitivity and specificity of BRAF molecular testing methods, this is unlikely to have contributed to any observed differences in results.^26^ In 2022, NICE provided guidance that BRAF-V600E IHC should be used as the first-line test to detect BRAF mutations, followed by PCR of IHC-negative melanomas.^2,26^ There was no equivalent *BRAF* test method guidance for our cohort from 2016 to 2021 and most laboratories used PCR testing, which had been well established since 2011, rather than IHC, which was less well studied then and expensive.^26,27^ This was confirmed by the small proportion of pathology reports that described BRAF IHC testing. This ameliorates the possibility that the BRAF-mutated group in this study represented a cohort of people who were IHC negative and PCR positive; however, it remains possible that some BRAF IHC reports were not reported to NDRS and so may be missing. The implementation of the National Genomic Test Directory for Cancer in England and access for all patients to NGS panels may provide greater information on the incidence of non-BRAF-V600E mutations, as well as data for *NRAS* and *KIT*. As characteristics and survival by genotype were explored in the *BRAF*-tested cohort, this cohort may not be generalizable to the population of patients with melanoma who did not undergo testing.

In conclusion, this study increases our understanding of the epidemiology of *BRAF* mutations in melanoma. The identified disparities in *BRAF* mutation testing indicate a need for public health initiatives to bridge these gaps with policy interventions and evidence-based guidelines. The association between *BRAF* mutations and lower survival, particularly for stage II melanoma, underscores the importance of incorporating *BRAF* testing into early-stage melanoma care.

## Supplementary Material

ljaf351_Supplementary_Data

## Data Availability

The raw data that support the findings of this study are available through an approved data request with NHS England’s Data Access Request Service.
