# Correction to: A national cohort study of melanoma *BRAF* status, testing patterns, patient and tumour characteristics, treatment and survival in England from 2016 to 2021

**DOI:** 10.1093/bjd/ljag197

**Published:** 2026-05-21

This is a correction to: Khaylen Mistry, Polly Jeffrey, Nick J Levell, Oliver Kennedy, Kathryn Richardson, Paul Craig, Chloe Bright, Siobhan Taylor, John Ragan, Dimitrios Karponis, Joanna Pethick, Katrina Lavelle, Fiona McRonald, Steven Hardy, Sally Vernon, Paul Lorigan, Zoe C Venables, A national cohort study of melanoma *BRAF* status, testing patterns, patient and tumour characteristics, treatment and survival in England from 2016 to 2021, *British Journal of Dermatology*, Volume 193, Issue 6, December 2025, Pages 1146–1154, https://doi.org/10.1093/bjd/ljaf351

In the originally published manuscript, the ‘**Results**’ section of the Abstract contained an error that has now been corrected. The sentence is emended to read:

“Patients with BRAF mutations had a lower 5-year net survival [55.9% (95% CI 52.7–59.2) vs. BRAF wildtype 5-year net survival 62.2% (95% CI 60.1-64.5)], particularly in stage II disease”.

Instead of:

“Patients with BRAF mutations had a lower 5-year net survival [55.9% (95% CI 52.7–59.2) vs. BRAF wildtype 5-year net survival 66.5% (95% CI 62.1–60.1)], particularly in stage II disease”.

Additionally, under the ‘**Survival by *BRAF* genotype**’ heading, a sentence has been emended to read:

“BRAF-mutated tumours had a lower net survival [BRAF wildtype 5-year net survival 62.2% (95% CI 60.1-64.5) vs. BRAF-mutated 5-year net survival 55.9% (95% CI 52.7– 59.2)], particularly in stage II disease [BRAF wildtype 5-year net survival 66.8% (95% CI 63.7–70.2) vs. BRAF-mutated 5-year net survival 55.5% (95% CI 50.5–61.1)] [Figure 1; Table S10 (see Supporting Information)]”

Instead of:

“BRAF-mutated tumours had a lower net survival [BRAF wildtype 5-year net survival 66.5% (95% CI 62.1–60.1) vs. BRAF-mutated 5-year net survival 55.9% (95% CI 52.7– 59.2)], particularly in stage II disease [BRAF wildtype 5-year net survival 66.8% (95% CI 63.7–70.2) vs. BRAF-mutated 5-year net survival 55.5% (95% CI 50.5–61.1)] [Figure 1; Table S10 (see Supporting Information)]”.

