# Supplementary material for: A national cohort study of melanoma BRAF status, testing patterns, patient and tumour characteristics, treatment and survival in England from 2016 to 2021
Source: Br J Dermatol. 2025 Sep 3;193(6):1146–54. doi: 10.1093/bjd/ljaf351 (PMC12626136; doi:10.1093/bjd/ljaf351)
Supplement: ljaf351_Supplementary_Data [file ljaf351_supplementary_data.docx]

**Supplementary Material**

**Table S1 – classification of melanoma**

|  | ICD-10 site code | ICD-O-3 morphology code | ICD-O-3 behavior code |
| --- | --- | --- | --- |
| Melanoma | C43^a^, C510-512, C518, C519, C600-C602, C608, C609, C632, C000-C002, C006, C009 | 8720-8780^b^ | >2^c^ |

^a^ C43 = melanoma, C510 = labium majus, C511 = labium minus, C512 = clitoris, C518 = overlapping lesion of vulva, C519 = vulva unspecified, C600 = prepuce of penis, C601 = glans of penis, C602 = body of penis, C609 = penis unspecified, C632 = scrotum, C000 = external upper lip, C001 = external lower lip, C002 = external lip unspecified, C006 = commissure of lip, C009 = lip unspecified.

^b^ Morphology codes for naevi and melanomas

^c^ Behavior code >2 = 3 (malignant, primary site), 6 (malignant, metastatic site), 9 (malignant, uncertain site)

**Table S2 – Definitions of co-variates**

| Variate | Definition |
| --- | --- |
| Molecular genetics | Molecular genetics data included the name of the gene tested, date the gene was tested, whether the test was abnormal, mutation subtype and the time between the pathological diagnosis date and the test for BRAF status date. |
| Ethnicity* | Ethnicity data are recorded in secondary care from derived datasets and captured by the National Disease Registration Service (NDRS) from Cancer Outcomes and Services Dataset (COSD), Patient Administration System (PAS), the National Radiotherapy Dataset (RTDS) and Diagnostic Imaging Dataset (DID). If no ethnicity record can be extracted from the preceding data sources, an annual additional search is performed using Hospital Episode Statistics (HES) records. Where two conflicting ethnicity values are found, the NDRS record is updated with the most recently recorded known ethnicity. 18 ethnicity categories +1 ‘not stated’ category are defined by Office for National Statistics for the decennial census and used by NDRS. Ethnicity data in HES and census are self-reported. This systematic approach ensures ethnicity data is complete and reproducible. Ethnicity was grouped into white, non-white (Asian (excluding Chinese), Black, Chinese, mixed, other) and unknown. |
| Skin site* | Skin site was grouped into head/neck (lip, eyelid, ear, face, scalp/neck), limbs (upper and lower), trunk, overlapping (multiple cutaneous sites)/unknown/external genitals. |
| All site | All site data was grouped into skin (skin, lips, external genitals (scrotum, penile, vulva, labial, clitoris)), mucosal (nasal cavity, accessory sinuses, nasopharynx, oral, oesophageal, duodenum, rectal, anal, vaginal, cervical), ocular, other (lung, central nervous system, urinary tract, endocrine). |
| Deprivation | Deprivation quintiles were calculated using the Index of Multiple Deprivation equal Lower Super Output Area of residence (small geographical unit) weighted deprivation measures at the time of diagnosis. |
| Charlson co-morbidity score | Charlson co-morbidity score was defined by the summed cancer and non-cancer component from Hospital Episode Statistics (HES) data with a look back period of 3 to 27 months from cancer diagnosis date. |
| Geographical region | Geography was defined as the government office region of residence at the time of diagnosis. There are 9 regions in England. |
| Stage | The majority of tumours were staged in Union for International Cancer Control (UICC) 8 or (American Joint Committee on Cancer) AJCC 7, with a small number in earlier versions of UICC or AJCC. |
| Previous cancer | Previous cancer was defined using International Classification of Diseases (ICD)-10 site codes and ICD-O3 morphology and behavior codes as any cancer (excluding non-melanoma skin cancer) within five years prior to melanoma diagnosis. |
| Surgery | Surgical codes were retrieved from the HES and the National Cancer Registration Dataset (NCRD) > -30 days from the melanoma diagnosis. Definitive surgery included wide local excision, Moh’s micrographic surgery and other complex excisions such as flaps or grafts. |
| Radiotherapy | Radiotherapy (> -30 days from the melanoma diagnosis) data were derived from Radiotherapy Dataset (RTDS). |
| Systemic therapy | Systemic Anti-Cancer Therapy (SACT) (> -30 days from the melanoma diagnosis) data were derived from the SACT dataset. SACT encompassed immunotherapy and targeted therapy. SACT regimens were combined so that a patient was counted once. SACT intent coding were not reported due to incompleteness and known inconsistencies in coding. |

* Grouping site and ethnicity data ensured sufficient counts to maintain anonymity and stabilise the multivariate models whilst maintaining an acceptable categorisation in line with current literature and clinical expertise.^28,29^

**Table S3 – Number of cutaneous melanomas BRAF tested per year**

| **Year** | **BRAF tested** |
| --- | --- |
| 2016 | 2083 |
| 2017 | 2097 |
| 2018 | 2216 |
| 2019 | 2357 |
| 2020 | 1955 |
| 2021 | 1782 |
| 2016-2021 | 12490 |

**Table S4 - Logistic regression of the association of stage at diagnosis with the time between the pathological diagnosis date and the test for BRAF status date (BRAF tested within 180 days of diagnosis)**

| **Stage** | **Tested <180d, n = 9720, n (%)** | **Tested** $\boldsymbol{\geq}$**180d, n = 2770, n (%)** | **Total, n = 12490, n (%)** | **Logistic regression, multivariate analysis, n = 12490, OR* (95% CI)** |
| --- | --- | --- | --- | --- |
| **I** | 760 (7.8) | 606 (21.9) | 1366 (10.9) | **2.00 (1.75-2.27)** |
| **II** | 3158 (32.5) | 1283 (46.3) | 4441 (35.6) | REF |
| **III** | 2878 (29.6) | 456 (16.5) | 3334 (26.7) | **0.35 (0.31-0.39)** |
| **IV** | 1178 (12.1) | 46 (1.7) | 1224 (9.8) | **0.11 (0.08-0.15)** |
| **Unknown** | 1746 (18.0) | 379 (13.7) | 2125 (17.0) | **0.77 (0.67-0.89)** |

*Odds ratio are for not BRAF tested within 180 days of diagnosis (reference is tested within 180 days). Multivariate model includes age, gender, site, ethnicity, deprivation, geographic region and stage.

**Table S5 – Stage breakdown of cutaneous melanoma by geographic region**

| **Region** | **I** | **II** | **III** | **IV** | **Unknown** | **Total** | **% Stage III/IV** |
| --- | --- | --- | --- | --- | --- | --- | --- |
| London | 4114 | 1067 | 404 | 214 | 1406 | 7205 | 8.6 |
| East of England | 5649 | 2022 | 902 | 312 | 1065 | 9950 | 12.2 |
| North-East | 3570 | 781 | 323 | 114 | 477 | 5265 | 8.3 |
| North-West | 7765 | 2229 | 1032 | 327 | 943 | 12296 | 11.1 |
| Yorkshire and the Humber | 4715 | 1417 | 711 | 216 | 701 | 7760 | 11.9 |
| East Midlands | 4024 | 1344 | 427 | 203 | 1067 | 7065 | 8.9 |
| West Midlands | 4645 | 1809 | 596 | 216 | 635 | 7901 | 10.3 |
| South-East | 10575 | 3606 | 1012 | 487 | 1968 | 17648 | 8.5 |
| South-West | 7296 | 2659 | 919 | 316 | 1263 | 12363 | 10.0 |

**Table S6 – Logistic regression of co-variates associated with BRAF testing**

| **Variable** | **Not tested, n = 74963 (85.7%)** | **BRAF tested , n = 12490 (14.3%)** | **Total, n = 87453** | **Logistic regression, multivariable analysis, n = 87453, OR* (95% CI)** |
| --- | --- | --- | --- | --- |
| **Gender** |  |  |  |  |
| Male | 36812 (49.1) | 7344 (58.8) | 44156 (50.5) | REF |
| Female | 38151 (50.9) | 5146 (41.2) | 43297 (49.5) | **0.82 (0.79-0.86)** |
| **Age band** |  |  |  |  |
| <70 | 43588 (58.1) | 6080 (48.7) | 49668 (56.8) | REF |
| 70-79 | 17949 (23.9) | 3635 (29.1) | 21584 (24.7) | 1.05 (1.00-1.11) |
| >=80 | 13426 (17.9) | 2775 (22.2) | 16201 (18.5) | **0.88 (0.83-0.93)** |
| **Site*** |  |  |  |  |
| Skin | 74963 (95.6) | 12490 (96.0) | 87453 (95.7) | REF |
| Mucosal | 347 (0.4) | 392 (3.0) | 739 (0.8) | **2.39 (2.04-2.80)** |
| Ocular | 3040 (3.9) | 82 (0.6) | 3122 (3.4) | **0.06 (0.05-0.08)** |
| Other | 53 (0.07) | 48 (0.4) | 101 (0.1) | **2.53 (1.67-3.82)** |
| **Skin site** |  |  |  |  |
| Head/neck | 13093 (17.5) | 2315 (18.5) | 15408 (17.6) | 0.99 (0.93-1.05) |
| Limbs | 36020 (48.1) | 5192 (41.6) | 41212 (47.1) | REF |
| Trunk | 24082 (32.1) | 3564 (28.5) | 27646 (31.6) | 0.97 (0.92-1.02) |
| Overlapping/unknown | 1768 (2.4) | 1419 (11.4) | 3187 (3.6) | **2.31 (2.11-2.53)** |
| /external genitals |  |  |  |  |
| **Ethnicity** |  |  |  |  |
| White | 68390 (91.2) | 11759 (94.1) | 80149 (91.6) | REF |
| Non-white | 866 (1.2) | 201 (1.6) | 1067 (1.2) | 1.15 (0.95-1.36) |
| Unknown | 5707 (7.6) | 530 (4.2) | 6237 (7.1) | **0.80 (0.72-0.89)** |
| **Deprivation quintile** |  |  |  |  |
| 1 (most deprived) | 7617 (10.2) | 1633 (13.1) | 9250 (10.6) | **1.25 (1.16-1.35)** |
| 2 | 11355 (15.1) | 2013 (16.1) | 13368 (15.3) | **1.13 (1.05-1.21)** |
| 3 | 15637 (20.9) | 2740 (21.9) | 18377 (21.0) | **1.17 (1.10-1.25)** |
| 4 | 18682 (24.9) | 3012 (24.1) | 21694 (24.8) | **1.11 (1.05-1.18)** |
| 5 (least deprived) | 21672 (28.9) | 3092 (24.8) | 24764 (28.3) | REF |
| **Stage** |  |  |  |  |
| 1 | 50987 (68.0) | 1366 (10.9) | 52353 (59.9) | REF |
| 2 | 12403 (16.5) | 4441 (35.6) | 16844 (19.3) | **13.67 (12.81-14.61)** |
| 3 | 2992 (4.0) | 3334 (26.7) | 6326 (7.2) | **42.28 (39.25-45.57)** |
| 4 | 1181 (1.6) | 1224 (9.8) | 2405 (2.8) | **31.37 (28.29-34.79)** |
| Unknown | 7400 (9.9) | 2125 (17.0) | 9525 (10.9) | **9.77 (9.03-10.56)** |
| **Region** |  |  |  |  |
| London | 6351 (8.5) | 854 (6.8) | 7205 (8.2) | 1.01 (0.92-1.11) |
| East of England | 8575 (11.4) | 1375 (11.0) | 9950 (11.4) | **1.09 (1.00-1.18)** |
| North-East | 4640 (6.2) | 625 (5.0) | 5265 (6.0) | **1.25 (1.12-1.39)** |
| North-West | 9906 (13.2) | 2390 (19.1) | 12296 (14.1) | **2.21 (2.05-2.39)** |
| Yorkshire and the Humber | 6904 (9.2) | 856 (6.9) | 7760 (8.9) | **0.85 (0.77-0.93)** |
| East Midlands | 6109 (8.1) | 956 (7.7) | 7065 (8.1) | **1.19 (1.08-1.30)** |
| West Midlands | 6118 (8.2) | 1783 (14.3) | 7901 (9.0) | **2.60 (2.39-2.82)** |
| South-East | 15662 (20.9) | 1986 (15.9) | 17648 (20.2) | REF |
| South-West | 10698 (14.3) | 1665 (13.3) | 12363 (14.1) | **1.17 (1.08-1.26)** |

*Odds ratio are for BRAF tested (reference not BRAF tested). Multivariate model includes gender, age, ethnicity, cutaneous site only, deprivation, stage, geographic region. A separate model was run for all sites (including mucosal, ocular and other). For this separate model n = 91415 (Not BRAF tested = 78403, BRAF tested = 13012)

**Table S7 - BRAF mutation subtypes for BRAF tested cutaneous melanoma**

| **BRAF mutation subtype** | **Tumour count (%), n=12490** |
| --- | --- |
| Wild-type | 8089 (64.8) |
| Mutant | 4401 (35.2) |
| V600E | 2099 (47.7) |
| BRAF mutated but mutation unknown | 868 (19.7) |
| V600X | 712 (16.2) |
| V600K | 495 (11.2) |
| Other | 119 (2.7) |
| V600R | 75 (1.7) |
| K601E | 33 (0.7) |

**Table S8 – Odds ratios for the association between co-variates and mutated BRAF genotype stratified by gender**

| **Males** | | | | |
| --- | --- | --- | --- | --- |
| **Variable** | **Wild type (WT), n = 4850 (66.0%)** | **Mutated, n = 2494 (34.0%)** | **Total, n = 7344** | **Logistic regression, multivariate analysis, n = 7344, OR* (95% CI)** |
| **Age (years)** |  |  |  |  |
| <70 | 1883 (38.8) | 1510 (60.5) | 3393 (46.2) | REF |
| 70-79 | 1667 (34.4) | 658 (26.4) | 2325 (31.7) | **0.51 (0.46-0.58)** |
| $\geq$80 | 1300 (39.2) | 326 (13.1) | 1626 (22.1) | **0.38 (0.32-0.43)** |
| **All Site**** |  |  |  |  |
| Skin | 4850 (95.8) | 2494 (99.6) | 7344 (97.1) | REF |
| Mucosal | 143 (2.8) | 6 (0.2) | 149 (2.0) | **0.08 (0.03-0.17)** |
| Ocular | 42 (0.8) | 1 (0.03) | 43 (0.6) | **0.04 (0.002-0.17)** |
| Other | 27 (0.5) | 3 (0.1) | 30 (0.4) | **0.20 (0.05-0.57)** |
| **Skin site** |  |  |  |  |
| Head/neck | 1351 (27.9) | 392 (15.7) | 1743 (23.7) | **0.41 (0.36-0.47)** |
| Lower limb | 735 (15.2) | 337 (13.5) | 1072 (14.6) | **0.53 (0.45-0.61)** |
| Upper limb | 860 (17.7) | 311 (12.5) | 1171 (15.9) | **0.44 (0.38-0.52)** |
| Trunk | 1346 (27.8) | 1163 (46.6) | 2509 (34.2) | REF |
| Overlapping/unknown | 558 (11.5) | 291 (11.7) | 849 (11.6) | **0.55 (0.45-0.66)** |
| /external genitals |  |  |  |  |
| **Ethnicity** |  |  |  |  |
| White | 4581 (94.4) | 2353 (94.3) | 6934 (94.4) | REF |
| Non-white | 80 (1.6) | 28 (1.1) | 108 (1.5) | **0.51 (0.32-0.79)** |
| Unknown | 189 (3.9) | 113 (4.5) | 302 (4.1) | 1.04 (0.81-1.33) |
| **Deprivation quintile** |  |  |  |  |
| 1 (most deprived) | 584 (12.0) | 341 (13.7) | 925 (12.6) | 1.18 (0.99-1.41) |
| 2 | 765 (15.8) | 414 (16.6) | 1179 (16.1) | 1.10 (0.93-1.29) |
| 3 | 1047 (21.6) | 548 (22.0) | 1595 (21.7) | 1.11 (0.95-1.28) |
| 4 | 1201 (24.8) | 601 (24.1) | 1802 (25.1) | 1.05 (0.91-1.21) |
| 5 (least deprived) | 1253 (25.8) | 590 (23.7) | 1843 (25.1) | REF |
| **Stage** |  |  |  |  |
| 1 | 434 (8.9) | 255 (10.2) | 689 (9.4) | **1.34 (1.11-1.61)** |
| 2 | 1930 (39.8) | 703 (28.2) | 2633 (35.9) | REF |
| 3 | 1125 (23.2) | 830 (33.2) | 1955 (26.6) | **1.63 (1.43-1.86)** |
| 4 | 487 (10.0) | 300 (12.0) | 787 (10.7) | **1.53 (1.27-1.84)** |
| Unknown | 874 (18.0) | 406 (16.3) | 1280 (17.4) | **1.21 (1.02-1.42)** |
| **Region** |  |  |  |  |
| London | 310 (6.4) | 185 (7.0) | 495 (6.7) | **1.29 (1.03-1.62)** |
| East of England | 520 (10.7) | 295 (11.5) | 815 (11.1) | 1.17 (0.96-1.41) |
| North-East | 227 (4.7) | 119 (5.7) | 346 (4.7) | 1.06 (0.82-1.37) |
| North-West | 930 (19.2) | 464 (19.0) | 1394 (19.0) | REF |
| Yorkshire and the Humber | 340 (7.0) | 182 (6.8) | 522 (7.1) | 1.03 (0.82-1.28) |
| East Midlands | 356 (7.3) | 221 (8.1) | 577 (7.9) | **1.27 (1.03-1.57)** |
| West Midlands | 705 (14.5) | 328 (13.2) | 1033 (14.1) | 0.94 (0.78-1.12) |
| South-East | 812 (16.7) | 369 (15.5) | 1181 (16.1) | 1.08 (0.91-1.29) |
| South-West | 650 (13.4) | 331 (13.3) | 981 (13.4) | 1.09 (0.91-1.31) |
| **Females** | | | | |
| **Variable** | **Wild type (WT), n = 3239 (62.9%)** | **Mutated, n = 1907 (37.1%)** | **Total, n = 5146** | **Logistic regression, multivariate analysis, n = 5146, OR* (95% CI)** |
| **Age (years)** |  |  |  |  |
| <70 | 1412 (43.6) | 1275 (66.9) | 2687 (52.2) | REF |
| 70-79 | 915 (28.2) | 395 (20.7) | 1310 (25.5) | **0.50 (0.43-0.58)** |
| $\geq$80 | 912 (28.2) | 237 (12.4) | 1149 (22.3) | **0.33 (0.28-0.40)** |
| **All Site**** |  |  |  |  |
| Skin | 3239 (91.9) | 1907 (99.3) | 5146 (94.5) | REF |
| Mucosal | 235 (6.7) | 8 (0.4) | 243 (4.4) | **0.06 (0.03-0.12)** |
| Ocular | 37 (1.0) | 2 (0.1) | 39 (0.7) | **0.09 (0.01-0.29)** |
| Other | 15 (0.4) | 3 (0.2) | 18 (0.3) | 0.40 (0.09-1.24) |
| **Skin site** |  |  |  |  |
| Head/neck | 412 (12.7) | 160 (8.4) | 572 (11.1) | **0.48 (0.38-0.60)** |
| Lower limb | 1186 (36.6) | 726 (38.1) | 1912 (37.2) | **0.65 (0.55-0.76)** |
| Upper limb | 735 (22.7) | 302 (15.8) | 1037 (20.2) | **0.43 (0.36-0.52)** |
| Trunk | 503 (15.5) | 552 (28.9) | 1055 (20.5) | REF |
| Overlapping/unknown | 403 (12.4) | 167 (8.8) | 570 (11.1) | **0.42 (0.33-0.54)** |
| /external genitals |  |  |  |  |
| **Ethnicity** |  |  |  |  |
| White | 3034 (93.7) | 1791 (93.9) | 4825 (93.8) | REF |
| Non-white | 61 (1.9) | 32 (1.7) | 93 (1.8) | 0.87 (0.54-1.36) |
| Unknown | 144 (4.4) | 84 (4.4) | 228 (4.4) | 0.93 (0.70-1.25) |
| **Deprivation quintile** |  |  |  |  |
| 1 (most deprived) | 434 (13.4) | 274 (14.4) | 708 (13.8) | 0.98 (0.80-1.21) |
| 2 | 524 (16.2) | 310 (16.3) | 834 (16.2) | 0.95 (0.78-1.15) |
| 3 | 734 (22.7) | 411 (21.6) | 1145 (22.3) | 0.96 (0.80-1.14) |
| 4 | 753 (23.2) | 457 (24.0) | 1210 (23.5) | 1.06 (0.89-1.26) |
| 5 (least deprived) | 794 (24.5) | 455 (23.9) | 1249 (24.3) | REF |
| **Stage** |  |  |  |  |
| 1 | 378 (11.7) | 299 (15.7) | 677 (13.2) | **1.48 (1.22-1.79)** |
| 2 | 1264 (39.0) | 544 (28.5) | 1808 (35.1) | REF |
| 3 | 754 (23.3) | 625 (32.8) | 1379 (26.8) | **1.58 (1.36-1.85)** |
| 4 | 276 (8.5) | 161 (8.4) | 437 (8.5) | **1.36 (1.08-1.72)** |
| Unknown | 567 (17.5) | 278 (14.6) | 845 (16.4) | **1.26 (1.03-1.53)** |
| **Region** |  |  |  |  |
| London | 238 (7.3) | 121 (6.3) | 359 (7.0) | 0.95 (0.72-1.24) |
| East of England | 350 (10.8) | 210 (11.0) | 560 (10.9) | 1.06 (0.84-1.33) |
| North-East | 147 (4.5) | 132 (6.9) | 279 (5.4) | **1.54 (1.16-2.04)** |
| North-West | 626 (19.3) | 370 (19.4) | 996 (19.4) | REF |
| Yorkshire and the Humber | 218 (6.7) | 116 (6.1) | 334 (6.5) | 0.92 (0.70-1.20) |
| East Midlands | 245 (7.6) | 134 (7.0) | 379 (7.4) | 0.99 (0.76-1.28) |
| West Midlands | 496 (15.3) | 254 (13.3) | 750 (14.6) | 0.88 (0.72-1.09) |
| South-East | 494 (15.3) | 311 (16.3) | 805 (15.6) | **1.29 (1.05-1.56)** |
| South-West | 425 (13.1) | 259 (13.6) | 684 (13.3) | 1.17 (0.94-1.44) |

*Odds ratio are for BRAF mutated (reference BRAF WT). Multivariate model includes gender, age, ethnicity, cutaneous site only, deprivation, stage, geographic region.

**A separate model for all sites (including mucosal, ocular and other). For this separate model n = 7566 for males and n = 5446 for females as it included non-cutaneous tumours.

**Table S9 – SACT received by genotype and stage**

| **Stage** | **% treated with targeted and immunotherapy** | | **% treated with targeted only** | | **% treated with immunotherapy only** | | **% treated with neither** | |
| --- | --- | --- | --- | --- | --- | --- | --- | --- |
|  | **BRAF wild-type (WT)** | **BRAF mutated** | **BRAF WT** | **BRAF mutated** | **BRAF WT** | **BRAF mutated** | **BRAF WT** | **BRAF mutated** |
| **1** | 0.7 (5/762) | 11.4 (61/534) | 0.4 (3/762) | 15.5 (83/534) | 30.6 (233/762) | 13.7 (73/534) | 68.4 (521/762) | 59.4 (317/534) |
| **2** | 0.4 (14/3149) | 14.3 (176/1232) | 0.5 (16/3149) | 17.9 (220/1232) | 31.8 (1002/3149) | 13.6 (168/1232) | 67.2 (2117/3149) | 54.2 (668/1232) |
| **3** | 0.6 (11/1859) | 18.1 (262/1446) | 0.6 (11/1859) | 31.1 (449/1446) | 59.0 (1096/1859) | 19.9 (288/1446) | 39.9 (741/1859) | 30.9 (447/1446) |
| **4** | 1.1 (8/752) | 26.8 (121/450) | 0.1 (1/752) | 29.8 (134/450) | 58.6 (441/752) | 18.7 (84/450) | 40.2 (302/752) | 24.7 (111/450) |
| **Unknown** | 0.5 (7/1412) | 17.1 (115/673) | 0.6 (9/1412) | 23.2 (156/673) | 39.4 (557/1412) | 19.0 (128/673) | 59.4 (839/1412) | 40.7 (274/673) |

**Table S10 – Five-year net survival by BRAF genotype, stage and gender**

| **Stage, BRAF, gender and age status** | **Total, n = 66709** | **5 year NS* (95% CI)** |
| --- | --- | --- |
| All stages BRAF wild-type (WT) | 6252 | 62.2 (60.1-64.5) |
| All stages BRAF mutated | 3405 | 55.9 (52.7-59.2) |
| All stages BRAF not tested | 57052 | 94.8 (94.1-95.5) |
| All stages BRAF WT males | 3717 | 59.3 (56.6-62.3) |
| All stages BRAF mutated males | 1916 | 51.9 (47.9-56.4) |
| All stages BRAF not tested males | 27751 | 93.5 (92.5-94.5) |
| All stages BRAF WT females | 2535 | 65.9 (62.5-69.4) |
| All stages BRAF mutated females | 1489 | 61.1 (56.4-66.1) |
| All stages BRAF not tested females | 29301 | 96.2 (95.2-97.3) |
| All stages BRAF WT 15-44 years | 345 | 76.4 (70.4-82.4) |
| All stages BRAF mutated 15-44 years | 510 | 77.4 (72.1-82.7) |
| All stages BRAF not tested 15-44 years | 8397 | 98.5 (98.1-98.9) |
| All stages BRAF WT 45-54 years | 571 | 68.8 (63.7-73.9) |
| All stages BRAF mutated 45-54 years | 562 | 66.2 (60.2-72.2) |
| All stages BRAF not tested 45-54 years | 8676 | 98.7 (98.3-99.1) |
| All stages BRAF WT 55-64 years | 943 | 67.8 (63.5-72.0) |
| All stages BRAF mutated 55-64 years | 700 | 62.3 (57.1-67.5) |
| All stages BRAF not tested 55-64 years | 10548 | 97.7 (97.1-98.3) |
| All stages BRAF WT 65-74 years | 1730 | 65.0 (61.5-68.4) |
| All stages BRAF mutated 65-74 years | 837 | 53.2 (47.8-58.5) |
| All stages BRAF not tested 65-74 years | 13735 | 97.0 (96.2-97.8) |
| All stages BRAF WT 75-99 years | 2663 | 50.8 (46.2-55.4) |
| All stages BRAF mutated 75-99 years | 796 | 46.4 (39.2-53.5) |
| All stage BRAF not tested 75-99 years | 15696 | 88.2 (86.1-90.3) |
| Stage 1 BRAF WT | 626 | 73.2 (66.3-80.9) |
| Stage 1 BRAF mutated | 442 | 69.2 (60.4-79.4) |
| Stage 1 BRAF not tested | 39854 | 102.5 (101.7-103.3) |
| Stage 2 BRAF WT | 2688 | 66.8 (63.7-70.2) |
| Stage 2 BRAF mutated | 1057 | 55.5 (50.5-61.1) |
| Stage 2 BRAF not tested | 9659 | 86.3 (84.7-87.9) |
| Stage 2A BRAF WT | 584 | 65.7 (59.5-72.6) |
| Stage 2A BRAF mutated | 275 | 56.8 (46.7-69.0) |
| Stage 2A BRAF not tested | 4305 | 95.8 (93.6-98.1) |
| Stage 2B BRAF WT | 996 | 71.1 (65.7-76.9) |
| Stage 2B BRAF mutated | 340 | 63.1 (55.2-72.2) |
| Stage 2B BRAF not tested | 3187 | 86.5 (83.5-89.5) |
| Stage 2C BRAF WT | 1095 | 62.1 (57.1-67.6) |
| Stage 2C BRAF mutated | 441 | 48.6 (41.4-57.0) |
| Stage 2C BRAF not tested | 2099 | 66.0 (62.0-70.2) |
| Stage 3 BRAF WT | 1530 | 58.5 (54.0-63.5) |
| Stage 3 BRAF mutated | 1200 | 60.4 (54.9-66.5) |
| Stage 3 BRAF not tested | 2074 | 72.0 (67.6-76.6) |
| Stage 4 BRAF WT | 623 | 34.5 (29.7-40.0) |
| Stage 4 BRAF mutated | 373 | 26.8 (20.3-35.4) |
| Stage 4 BRAF not tested | 855 | 34.0 (29.3-39.4) |
| Stage unknown BRAF WT | 785 | 63.9 (57.5-70.9) |
| Stage unknown BRAF mutated | 333 | 54.8 (44.8-66.9) |
| Stage unknown BRAF not tested | 4678 | 81.4 (77.9-85.2) |

*****Age-standardised net survival (NS) at 5 years was calculated by comparing overall survival from diagnosis date in patients with BRAF tested cutaneous melanoma between 2016-2020 compared to an age, year, gender, deprivation and geography matched general population cohort

**Table S11 - Univariate and multivariate Cox model for disease-specific death by BRAF genotype and co-variates**

| **Variable** | **Total, n = 9657 (100%)** | **Melanoma-specific death, n = 3185 (100%)** | **Cox regression, univariate analysis, n = 9657, HR* (95% CI)** | **Cox regression, multivariate analysis, n = 9657, HR* (95% CI)** |
| --- | --- | --- | --- | --- |
| **BRAF genotype** |  |  |  |  |
| Wild-type (WT) | 6252 (64.7) | 2003 (62.9) | REF | REF |
| Mutated | 3405 (35.3) | 1182 (37.1) | 1.06 (0.99-1.14) | **1.19 (1.10-1.28)** |
| **Gender** |  |  |  |  |
| Male | 5633 (58.3) | 2003 (62.9) | REF | REF |
| Female | 4024 (41.7) | 1182 (37.1) | **0.76 (0.71-0.82)** | **0.84 (0.78-0.91)** |
| **Age (years)** |  |  |  |  |
| <70 | 4756 (49.2) | 1320 (41.4) | REF | REF |
| 70-79 | 2776 (28.7) | 994 (31.2) | **1.47 (1.35-1.59)** | **1.47 (1.35-1.60)** |
| $\geq80$ | 2125 (22.0) | 871 (27.3) | **2.08 (1.91-2.27)** | **2.29 (2.08-2.51)** |
| **Skin site** |  |  |  |  |
| Head/neck | 1862 (19.3) | 569 (17.9) | **1.11 (1.01-1.23)** | 0.92 (0.83-1.02) |
| Limbs | 4259 (44.1) | 1261 (39.6) | REF | REF |
| Trunk | 2949 (30.5) | 1086 (34.1) | **1.33 (1.23-1.45)** | **1.30 (1.19-1.42)** |
| Overlapping/unknown | 587 (6.1) | 269 (8.4) | **2.21 (1.94-2.52)** | **1.19 (1.03-1.38)** |
| /external genitals |  |  |  |  |
| **Ethnicity** |  |  |  |  |
| White | 9065 (95.3) | 3036 (95.3) | REF | REF |
| Non-white | 156 (1.6) | 57 (1.8) | 1.10 (0.85-1.43) | 1.27 (0.97-1.66) |
| Unknown | 436 (4.5) | 92 (2.9) | **0.60 (0.49-0.74)** | **0.72 (0.58-0.89)** |
| **Deprivation quintile** |  |  |  |  |
| 1 (most deprived) | 1271 (13.2) | 468 (14.7) | **1.36 (1.21-1.53)** | **1.40 (1.25-1.58)** |
| 2 | 1562 (16.2) | 557 (17.5) | **1.26 (1.13-1.40)** | **1.31 (1.17-1.47)** |
| 3 | 2099 (21.7) | 675 (21.2) | 1.11 (1.00-1.23) | **1.13 (1.02-1.26)** |
| 4 | 2308 (23.9) | 766 (24.1) | **1.14 (1.03-1.26)** | **1.18 (1.07-1.31)** |
| 5 (least deprived) | 2417 (25.0) | 719 (22.6) | REF | REF |
| **Stage** |  |  |  |  |
| 1 | 1068 (11.1) | 245 (7.7) | **0.64 (0.55-0.73)** | **0.74 (0.64-0.85)** |
| 2 | 3745 (38.8) | 1170 (36.7) | REF | REF |
| 3 | 2730 (28.3) | 829 (26.0) | 0.97 (0.88-1.06) | 1.06 (0.97-1.17) |
| 4 | 996 (10.3) | 614 (19.3) | **3.40 (3.09-3.75)** | **3.47 (3.12-3.86)** |
| Unknown | 1118 (11.6) | 327 (10.3) | 1.01 (0.90-1.15) | **1.04 (0.92-1.18)** |
| **Region** |  |  |  |  |
| London | 656 (6.8) | 189 (5.9) | 0.90 (0.76-1.06) | 0.89 (0.76-1.06) |
| East of England | 1072 (11.1) | 379 (11.9) | **1.18 (1.04-1.35)** | **1.20 (1.05-1.37)** |
| North-East | 410 (4.2) | 159 (5.0) | **1.44 (1.21-1.72)** | **1.49 (1.25-1.78)** |
| North-West | 1833 (19.0) | 556 (17.5) | REF | REF |
| Yorkshire and the Humber | 650 (6.7) | 249 (7.8) | **1.29 (1.11-1.50)** | **1.22 (1.05-1.41)** |
| East Midlands | 762 (7.9) | 308 (9.7) | **1.44 (1.25-1.66)** | **1.50 (1.30-1.73)** |
| West Midlands | 1356 (14.0) | 423 (13.3) | 1.08 (0.95-1.23) | **1.17 (1.03-1.32)** |
| South-East | 1573 (16.3) | 461 (14.5) | 0.96 (0.85-1.09) | 0.99 (0.87-1.12) |
| South-West | 1345 (13.9) | 461 (14.5) | **1.15 (1.02-1.31)** | **1.16 (1.02-1.31)** |
| **Charlson score** |  |  |  |  |
| Mean (SD) | 0.39 (0.99) | 0.48 (1.09) | **1.20 (1.16-1.23)** | **1.13 (1.09-1.16)** |
| **Previous cancer** |  |  |  |  |
| No previous cancer | 9022 (93.4) | 2944 (92.4) | REF | **REF** |
| Previous cancer | 633 (6.6) | 241 (7.6) | **1.34 (1.17-1.53)** | 1.06 (0.92-1.22) |

*Hazard ratio is for disease-specific death defined as C43/C80 measuring survival from diagnosis date. Multivariate model includes gender, age, site, ethnicity, deprivation, stage, geographic region, Charlson score, previous cancer in past five years.

**Table S12 – Multivariate Cox model for disease-specific death by BRAF genotype where survival time starting from the diagnosis date and BRAF testing date**

| **Variable** | **Total, n = 5339 (100%)** | **Melanoma-specific death, n = 1661 (100%)** | **Cox regression, multivariate analysis, measuring survival from genetic test date, n = 5339, HR* (95% CI)** | **Cox regression, multivariate analysis, measuring survival from diagnosis date, n = 5339, HR* (95% CI)** |
| --- | --- | --- | --- | --- |
| **BRAF genotype** |  |  |  |  |
| Wild-type (WT) | 3529 (66.1) | 1055 (63.5) | REF | REF |
| Mutated | 1810 (33.9) | 606 (36.5) | **1.18 (1.06-1.31)** | **1.18 (1.06-1.31)** |

*Hazard ratio is for disease-specific death defined as C43/C80. Multivariate model includes gender, age, site, ethnicity, deprivation, stage, geographic region, Charlson score. Cohort restricted to pathological diagnosis date - test for BRAF status date ≤ 90 days.

**Table S13 – Multivariate Cox model for disease-specific death by SACT in BRAF mutated patients**

| **Variable** | **Total, n* = 1913 (100%)** | **Melanoma-specific death, n = 818 (100%)** | **Cox regression, multivariate analysis, n = 1913, HR** (95% CI)** |
| --- | --- | --- | --- |
| **Systemic therapy** |  |  |  |
| Immunotherapy only | 526 | 117 | **0.36 (0.29-0.45)** |
| Targeted therapy only | 792 | 353 | **REF** |
| Targeted and immunotherapy | 595 | 348 | **1.19 (1.02-1.39)** |

*BRAF mutated patients who received SACT with no previous cancer (excluding non-melanoma skin cancer)

**Hazard ratio is for disease-specific death defined as C43/C80 measuring survival from diagnosis date. Multivariate model includes gender, age, site, ethnicity, deprivation, stage, geographic region, Charlson score.

**Figure S1 – Breakdown of England by a) region (including population per region) and b) genomic laboratory hubs**

**
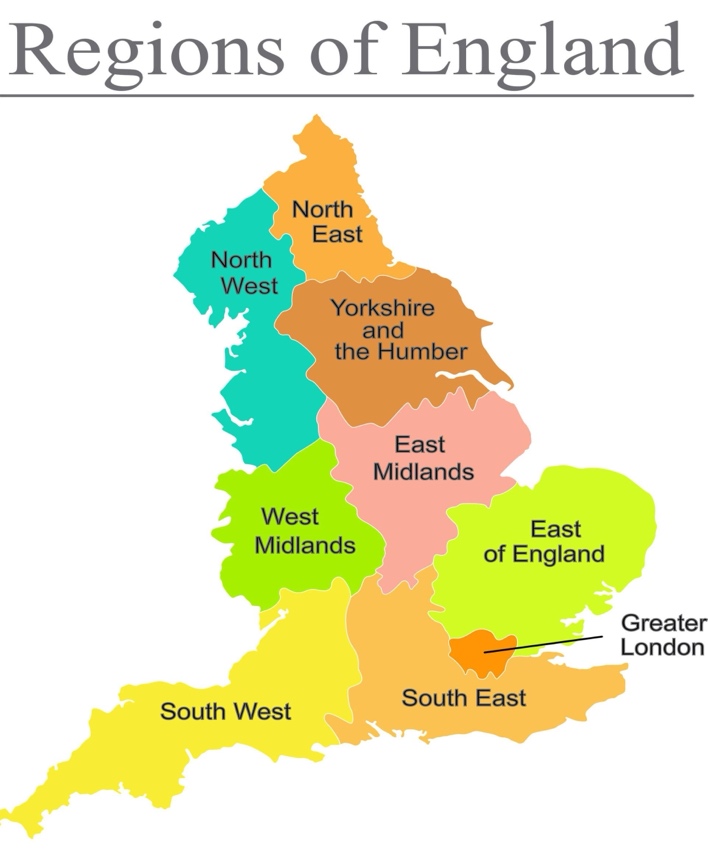

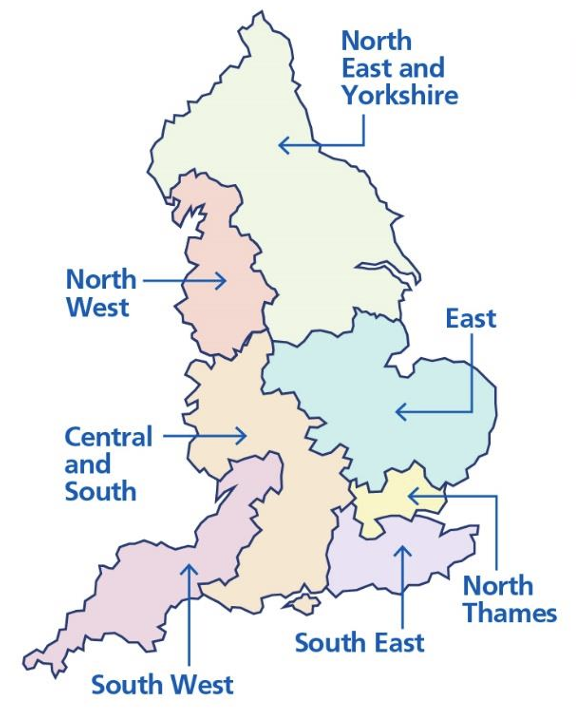
**

**b)**

**a)**

| **Region** | **Population*** |
| --- | --- |
| London | 8,833,300 |
| East of England | 6,237,400 |
| North-East | 2,629,400 |
| North-West | 7,316,100 |
| Yorkshire and the Humber | 5,443,900 |
| East Midlands | 4,815,200 |
| West Midlands | 5,889,300 |
| South-East | 9,150,200 |
| South-West | 5,609,700 |

*Population in 2018 according to Government Office of National Statistics data
